# Effects of Lipid Deposition on Viscoelastic Response in Human Hepatic Cell Line HepG2

**DOI:** 10.3389/fphys.2021.684121

**Published:** 2021-09-01

**Authors:** Rui Li, Yang Bu, Chendong Yang, Jizeng Wang

**Affiliations:** Key Laboratory of Mechanics on Disaster and Environment in Western China, Ministry of Education, College of Civil Engineering and Mechanics, Lanzhou University, Lanzhou, China

**Keywords:** liver steatosis, viscoelasticity, atomic force microscopy, oleic acid, HepG2 cells

## Abstract

Hepatic steatosis is associated with various liver diseases. The main pathological feature of steatosis is the excessive lipid accumulation. Ultrasound has been extensively used for the diagnosis of hepatic steatosis. However, most ultrasound-based non-invasive methods are still not accurate enough for cases with light lipid infiltration. One important reason is that the extent to which lipid infiltration may affect mechanical properties of hepatocytes remains unknown. In this work, we used atomic force microscope and *in vitro* dose-dependent lipid deposition model to detect the quantitative changes of mechanical properties under different degrees of steatosis in a single-cell level. The results show that hepatic cells with lipid deposition can be treated as linear viscoelastic materials with the power law creep compliance and relaxation modulus. Further analysis showed that even slight accumulation of lipid can lead to measurable decrease of stiffness and increased fluidity in liver cells. The accurate detection of viscoelastic properties of hepatocytes and the analysis methods may provide novel insights into hepatic steatosis grading, especially in the very early stage with reversible liver lesion. The application of viscoelasticity index for grading fat deposition might be a new detection indicator in future clinical diagnosis.

## Introduction

Hepatic steatosis is associated with various liver diseases (Sanyal et al., 2010; Hoyles et al., 2018). Some major diseases associated with steatosis include non-alcoholic fatty liver disease (NAFLD) and alcoholic liver disease (ALD) (Seitz et al., 2018; Gu et al., 2019), which are the major chronic liver diseases with a growing incidence every year. In severe cases, NAFLD can lead to liver dysfunction, hepatitis, and cirrhosis. An effective drug that can prevent and treat NAFLD has not been developed. Most patients with NAFLD may have no obvious clinical symptoms (Krawitz and Pyrsopoulos, 2020). Early control and intervention can effectively reverse disease progression. Thus, early diagnosis is highly important for patients with NAFLD.

Pathological diagnosis is the gold standard for confirming steatosis (Castera et al., 2019). This diagnosis procedure is invasive and is only used for the final determination for severe cases. However, early pathological changes usually occur in a certain part of the liver or dispersed in the liver. The recommended size for the biopsy is usually 1.5–3 cm (Rockey et al., 2009). The evaluation of focal lesions by invasive diagnosis is still affected by sampling error. Non-invasive imaging methods are widely used in clinical practice as alternative to liver biopsy (Lee and Park, 2014), and these methods include ultrasound (US), computed tomography, and magnetic resonance (MR) (Saadeh et al., 2002; Lee et al., 2010; Lee and Park, 2014; Zhang et al., 2014; Petäjä and Yki-Järvinen, 2016). The US diagnosis of hepatic steatosis is the most common imaging method, because it its widely used, safe, and inexpensive (Mazhar et al., 2009). However, US only has a reliable assessment in moderate and severe degree of steatosis (≥30%) but is not accurate for steatosis; it only meets the diagnostic criteria or is within mild degree (<20–30%; Dasarathy et al., 2009; Hernaez et al., 2011; Lee and Park, 2014). Therefore, high accuracy is still highly required in the US.

The inaccurate quantification of mild and local steatosis is related to the unclear changes in the mechanical properties of diseased liver tissue and the resolution of image feature recognition. The principle of US imaging method is based on the mechanical wave propagation in elastic media. Its propagation speed and amplitude are affected by the elastic modulus and bulk modulus of the medium. For viscoelastic (incomplete elasticity) media, the mechanical wave attenuates inside, and the attenuation speed is positively related to the viscosity of the medium (Righetti et al., 2002, 2003; Mueller, 2020b). Therefore, the US is an imaging method that is based on the differences in tissue mechanical properties. However, the imaging resolution is affected by input factors, such as wavelength, frequency, and wave constraint width. At present, the resolution of clinical device can only reach the millimeter level. Mild degree steatosis is limited to the lipid deposition in scattered cells, and reaching the resolution of the deposition area is difficult to reach, making US ineffective for the diagnosis of mild cases. The main pathology feature of liver steatosis is the excess lipid accumulation in liver. In mild cases, only local or small amount of lipid deposition is observed in hepatocytes. Therefore, the quantitative characterization of lipid deposition on the mechanical properties of liver tissue is needed for the accurate identification of mild fatty infiltration. How lipid deposition in liver cells affects the mechanical properties is essential for the accurate diagnosis of liver tissue lesions. A significant amount of work has been done in clinical practice through *in vivo* and *ex vivo* studies. Liver stiffness representing solid character has been studied as elastic moduli for years (Mueller and Sandrin, 2010). Yin et al. (2007) showed that steatosis has no effect on the stiffness of liver tissue via MRI measurements. They speculated that the stiffness range of fatty tissue is similar to the normal liver. Thus, the existence of hepatic steatosis in liver tissue has no obvious effect on the shear stiffness. A study measured by transient elastography on patients with ALD showed that steatosis is not related to liver stiffness (Rausch et al., 2016). Mueller (2020a) listed the stiffness of various living tissues quantified by elastography showed that the stiffness of fat tissue is about six times lower than that of the liver. Viscosity is another reference factor, suggesting that fat has an influence on mechanical properties. Barry et al. (2014) proved that fat adds viscosity to mouse livers and human liver samples. They suggested that the viscosity has a potential for steatosis scoring. Zhu et al. (2015) used gelatin-based phantoms containing a different ratio of castor oil to mimic different degrees of steatosis. Their results showed a viscosity increment with the addition of castor oil. However, a prospective clinical study using shear wave elastography found that viscosity has a remarkable effect with fibrosis rather than steatosis (Deffieux et al., 2015). Moreover, fat is a soft material with fluidity that lowers mechanical properties in liver tissue (Karlas and Mueller, 2020), which may help attenuate the harmful effects of mechanical energy generated by impulse waves (Mueller, 2016). Whether steatosis affects the mechanical properties of liver remains to be determined (Mueller et al., 2020).

From a perspective of materials, liver tissue is a composite material containing hepatocyte, hepatic sinusoid, perisinusoidal cells, and tissue matrix. The overall mechanical properties are related to each component as described in the sinusoidal pressure hypothesis (Mueller, 2016). However, only the hepatocytes are mainly affected by fat deposition. Thus, the mechanical properties of single hepatocyte affected by different degrees of fat depositions should be studied to quantitatively describe the relationship between mechanical properties changes of hepatic lobule and fat deposition. Furthermore, a reference value for the diagnostic criteria of fatty liver in precise localization and classifications should be provided.

Many attempts have been focused on the use of atomic force microscopy (AFM) to characterize the mechanical fingerprint of healthy, fibrosis, and malignant liver cells (Braet et al., 2018). Hepatoma cells with different metastasis ability showed disparity in terms of the distribution patterns of Young’s modulus (Tian et al., 2015). Differences in elastic modulus are found between normal liver, hepatoma, liver embryonic stem cells (Kim et al., 2013; Sun et al., 2016; Tsikritsis et al., 2016). An AFM topology study showed that the increased surface roughness and reduced cytoskeleton height are associated with curcumin-induced G2/M phase arrest in HepG2 cells (Jiang et al., 2013). These findings demonstrate the advantages of using AFM as a nanoscale measurement tool in capturing the biomechanical characteristics of liver cells. However, living cells are soft materials with rheological properties (Desprat et al., 2005). Hepatocytes are essential for whole-body energy metabolism and synthesis. The biosynthetic reaction is reflected by the composition change of cytoplasm rather than cytoskeletal structure, and this process might be difficult to capture based on transient elastic changes. Cytoplasmic component may not contribute to shear deformation of the cells, but it is highly incompressible. Considering that the cytoplasm is rich in different molecules such as lipids, carbohydrates, and proteins, dynamic changes in cell composition may be reflected in energy dissipation and time-dependent deformations. Our previous work has showed the viscoelastic differences between different cells in the liver (Bu et al., 2019). Therefore, the viscoelastic parameters may be used as indicators to distinguish cells with different lipid depositions.

The human hepatoma cell line HepG2 preserves part of the hepatic cell characteristics, and it has been widely used to study hepatocyte functions (Knowles et al., 1980). In this work, an oleic acid (OA)-induced hepatocyte deposition model with a gradient change was used to simulate intracellular lipid deposition. Following our previous study (Bu et al., 2019), we adopted a three-parameter power-law-type constitutive relation to fit the experimental measurements, and this method is better than the five-parameter classical spring-dashpot. The combination of the proposed power law expression and AFM indentation measurements on the creep compliance and relaxation modulus will provide a unique way in determining the viscoelastic properties of the HepG2 cells with different lipid deposition levels. Based on this technique, we expect that the experimental results can quantitatively reveal the dependence of viscoelastic properties of cells on their lipid accumulation.

## Materials and Methods

### Cell Lines and Cell Culture

Human hepatoma cell line HepG2 (Cell Bank of the Chinese Academy of Sciences, Shanghai, China) was cultured in Dulbecco’s modified Eagle’s medium (Gibco, Thermo Fisher Scientific, United States) containing 10% (v/v) fetal bovine serum (Gibco^TM^, Thermo Fisher Scientific, United States) supplemented with 100 U/ml penicillin and 100 μg/ml streptomycin in an incubator (INE800749L, Memmert, Germany) containing 5% CO_2_ at 37°C. Cells in logarithmic growth phase were used in the experiments.

### OA/BSA Complex Preparation

The OA/BSA complex solution was prepared as described before (Cousin et al., 2001; Yun et al., 2006). Briefly, 100 mM OA (Sigma-Aldrich, United States) solution was added to 10% fatty acid-free BSA (Solarbio, Beijing, China) stock solution. The mixture was incubated in a water bath for 30 min at 55°C to prepare a 5 mM OA/10% BSA complex. The solution was filter-sterilized through a 0.22-μm syringe filter (Millipore Corporation, Bedford, United States) after cooling to room temperature. The complex was used within 4 weeks.

### Proliferation Assay

The proliferation rate was evaluated by 3-(4,5-dimethylthiazol-2-yl)-2,5-diphenyltetrazolium bromide (MTT) assay (Sigma-Aldrich, MO, United States). HepG2 cells were seeded into a 96-well plate (3 × 10^3^ per well; Corning, NY, United States) and cultured for 24 h at 37°C. Then, DMEM containing different concentrations of FFA/BSA complex solution were added. After incubated for 24–72 h, 20 μL of MTT (5 mg/mL) was added to each well and incubated at 37°C for 4 h. The media was then removed, and 150 μL of dimethyl sulfoxide (Sigma-Aldrich, MO, United States) was added to each well to dissolve the crystal. The absorbance was measured at a wavelength of 570 nm by using a microplate reader (Infinite 200 PRO, TECAN, Switzerland).

### Oil Red O (ORO) Staining

Lipid droplets in cells were stained with ORO staining kit (Solarbio, Beijing, China). HepG2 cells were seeded on chamber slides. After treatment for 24 h, cells were fixed with 4% buffered paraformaldehyde for 30 min and washed for three times with PBS. The slides were stained according to the manufacturer’s instructions. Morphological changes were observed under light microscopy (Olympus, Tokyo, Japan). Positive area was measured using Image J software (Version 1.52, National Institutes of Health, United States). The extent of intracellular oil lipids was measured as described by Cui et al. (2010). ORO was extracted by isopropanol. The absorbance was measured at a wavelength of 405 nm by using a microplate reader (Infinite 200 PRO, TECAN, Switzerland).

### Immunofluorescent Staining

HepG2 cells were seeded on chamber slides. After treatment for 24 h, cells were fixed with 4% buffered paraformaldehyde for 30 min and washed thrice with PBS. For the detection of cell apoptosis, nuclei were labeled with Hoechst 33342 at 0.5 mg/mL for 15 min and washed with PBS. For actin cytoskeleton staining, cells were permeabilized with 0.1% Triton X-100 in PBS for 3 min and blocked with 1% BSA. The slides were stained with FITC-labeled Phalloidin (Sigma-Aldrich, MO, United States) for 1 h. Slides were sealed with ProLong glass antifade mountant (Thermo Fisher Scientific, MA, United States) and stored at room temperature for 24 h in the dark. The slides were observed under fluorescence microscope (Olympus, Tokyo, Japan). Fluorescence intensity was measured using Image J software (Version 1.52, National Institutes of Health, United States).

### AFM Indentation Assay

The AFM indentation assay and the theoretical model were conducted as previously described (Bu et al., 2019). Cells were seeded and treated in sterilized 35-mm petri dishes. A NanoWizard III AFM (JPK Instruments, Berlin, Germany) was used for the creep measurements of the cells. A silicon nitride AFM cantilever (NovaScan, Chicago, United States) with a polystyrene bead of 4.5 μm diameter was used. The spring constant was 0.01 N/m. The cantilever was approached to the cell at a velocity of 50 μm/s until the preset force was reached. Once a preset force for the creep was reached, the approaching was stopped by controlling the position of cantilever base. The force was kept constant for 10 s, and the cantilever retracted at a velocity of 1 μm/s. Each cell was only approached once. At least 60 individual cells were tested in each group.

### Theoretical Model and Data Processing

The hepatic cells are assumed as incompressible linear viscoelastic materials (Costa, 2003), and the stress–strain relationship can be defined through the creep compliance, *J* (*t*), which is expressed as follows:

(1) $$\varepsilon_{i\,j}\,\left(t\right)=\int_{0}^{t}J\,\left(t-\tau \right)\,\frac{d\,\sigma_{i\,j}\,\left(\tau \right)}{d\,\tau }\,d\tau .$$

Following Bu et al. (2019), we choose the power-law-type creep kernel function as follows:

(2) $$J\,\left(t\right)=\frac{1}{E_{0}}\,{\left(\frac{t}{\tau_{0}}\right)}^{\beta }$$

where *E*_0_ is the elastic modulus of viscoelastic material at time *τ*_0_, and *β* characterizes the degree of dissipation or “fluidity” of the material. If *β* approaches zero, then Equation (2) degrades into the Hook Law. If *β* approaches unity, then Equation (2) just corresponds to the Newtown fluid. In the following sections, we set *τ*_0_ to a very small timescale (∼10^−5^s), then *E*_0_ represents the instantaneous stiffness of the cell (Kollmannsberger and Fabry, 2011).

In AFM indentation tests, the tip of the AFM probe is considered as rigid sphere indenter, and the cells are treated as a viscoelastic half space. By neglecting the interfacial friction, the Hertz–Sneddon theory (Sneddon, 1965) is adopted to describe the indentation process. The relationship between the indentation depth, δ, and indentation force, *P*, can be written as (Yang, 1966) follows:

(3) $$\delta^{3/2}\,\left(t\right)=\frac{3}{8\,\sqrt{R}}\,\int_{0}^{t}J\,\left(t-\tau \right)\,\frac{d\,P\,\left(t\right)}{d\,\tau }\,d\tau$$

where *R* is the radius of polystyrene bead.

In our creep experiments, the indentation force can be pre-set as a step function as follows:

(4) $$P\,\left(t\right)=P_{0}\,\text{H}\,\left(t\right)$$

in which *P*_0_ is the amplitude of the loading force, and H (*t*) is the so-called Heaviside step function. By submitting Equation (4) into Equation (3), the measured creep function can be obtained as follows:

(5) $$J\,\left(t\right)=\left\langle \frac{8\,\sqrt{R}}{3\,P_{0}}\,\delta^{3/2}\,\left(t\right)\right\rangle$$

Notably, *J* (*t*) in Equation (5) is obtained by performing ensemble average on sufficiently large number of independent experiments.

**FIGURE 1:**
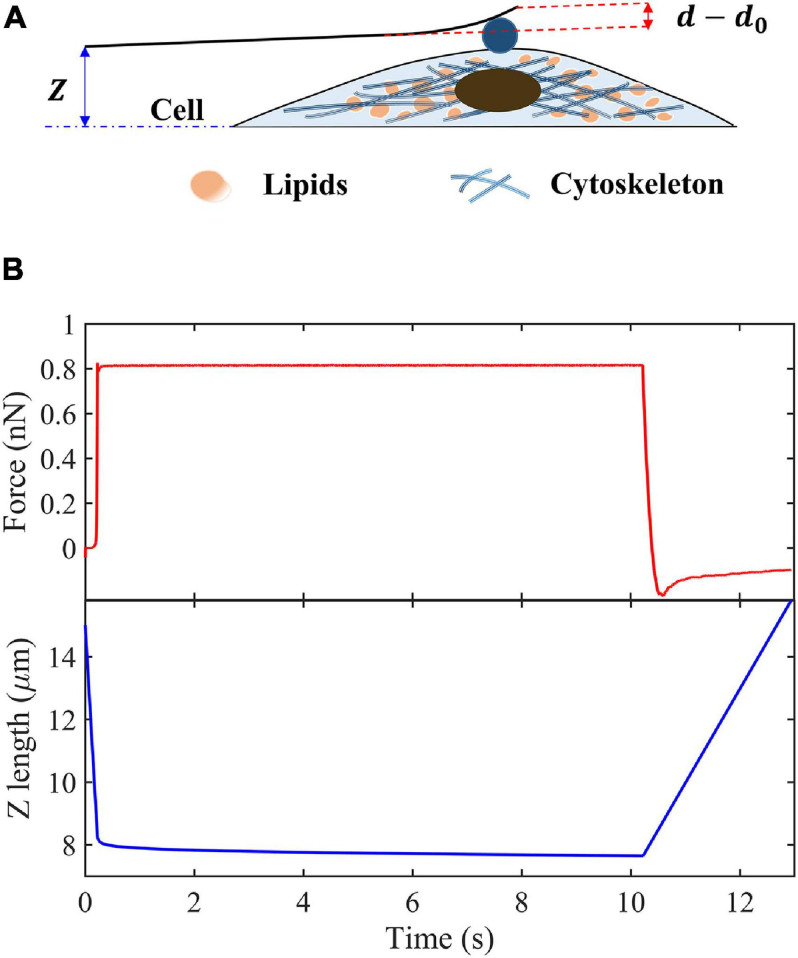
Schematic of creep experiments on living cells. **(A)** Schematic of the indentation experiment. **(B)** Indentation force and Z-position of the cantilever base in the creep measurements.

Figure 1 shows the process of a single-cell indentation. Contact point is defined as the moment when the indenter first contact with the surface of cells, where *Z* is the vertical height of cantilever base, and *Z*_0_ is the value of *Z* just at the contact point, *d* is the cantilever deflection at the location of tip, and *d*_0_ is its value at contact point. Then, the indentation depth δ can be expressed as follows:

(6) $$\delta =Z-Z_{0}-\left(d-d_{0}\right)$$

We assume that the cantilever is deformed like a Hookean spring with elastic constant, *k*. Then the indentation force and cantilever deflection can be related as follows:

(7) $$P=k\,\left(d-d_{0}\right).$$

where *d*′ = *P*_0_/*k* + *d*_0_. By keeping the indentation force constant, *P*_0_, the creep compliance can be obtained as follows:

(8) $$J\,\left(t\right)=\left\langle \frac{8\,\sqrt{R}}{3\,P_{0}}\,{\left[Z\,\left(t\right)-Z_{0}-d_{0}-d^{\prime }\right]}^{3/2}\right\rangle$$

### Statistical Analysis

All statistical analyses were performed using SPSS 26 (IBM, NY, United States) and GraphPad Prism software 7.0 (GraphPad Software, CA, United States). Data were expressed as means ± standard deviation. Statistical comparisons of the results were carried out using one-way ANOVA followed by the Bonferroni correction. *P*-value of < 0.05 was considered statistically significant.

## Results

### Toxicity of OA on HepG2 Cells

**FIGURE 2:**
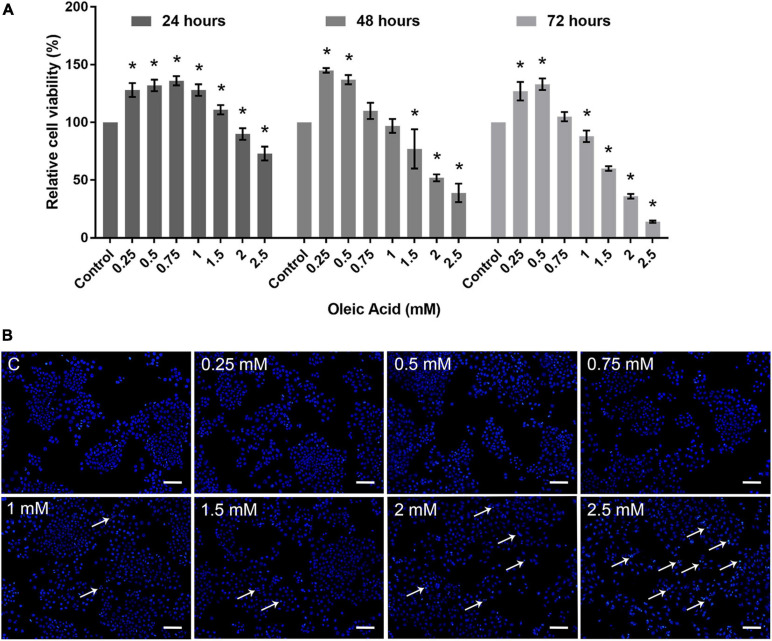
Toxicity of OA on HepG2 cells. **(A)** Proliferation ratio of HepG2 cells treated with different concentrations of OA for 24, 48, and 72 h. **(B)** The evaluation of apoptosis for 24 h. Hoechst 33342 was used to stain the nuclei. Apoptotic cells were indicated by white arrowheads. Images taken at 10 × magnification. Scale bars: 100 μm. Experiments were performed in triplicates in three independent experiments. *p < 0.05, compared with the control groups.

Cytotoxicity assay was carried out to determine the dose-dependent response of OA on HepG2 cells. As shown in Figure 2A, HepG2 cells exposed to OA concentration lower than 1.5 mM did not show proliferation inhibition after 24 h. Cells exposed to 2 and 2.5 mM reduced the viability rate at 24, 48, and 72 h significantly. Hoechst 33342 staining was used to detect apoptosis after 24 h (Figure 2B). Mitotic cells were incidentally detected. Cells exposed to OA concentration higher than 1.5 mM showed increased chromatin condensation, nuclear fragmentation, and apoptotic bodies after 24 h. The majority of cells nuclei morphology showed no apparent abnormality under 1.5 mM. Therefore, a concentration lower than 1.5 mM and time of 24 h were chosen as the optimal conditions to observe the dose-dependent effect of HepG2 cell lipid deposit.

### OA Increased Lipid Deposition on HepG2 Cells

**FIGURE 3:**
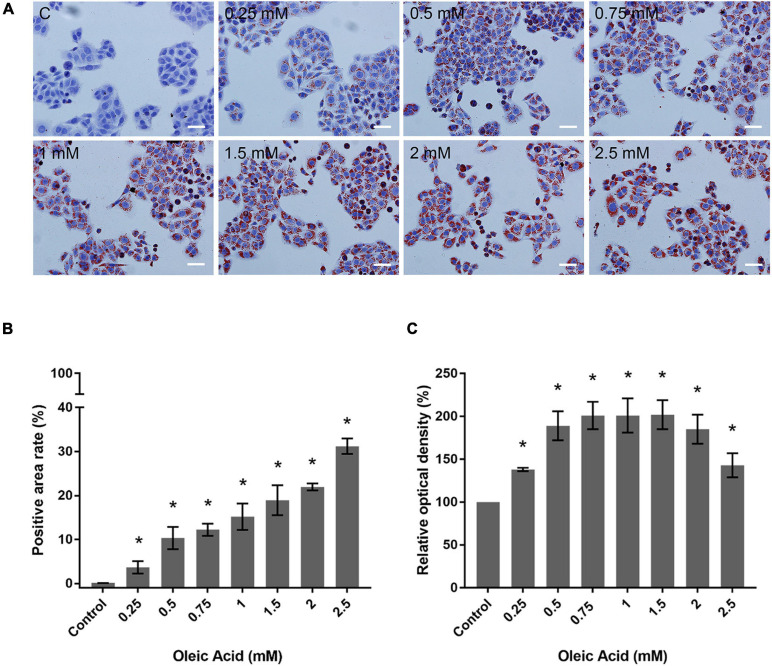
Effects of OA concentration on lipid accumulation in HepG2 cells. **(A)** ORO staining results of HepG2 cells treated with different concentrations of OA for 24 h. Red staining represents lipids. Images taken at 20 × magnification. Scale bars: 50 μm. **(B)** Positive staining area of ORO staining. **(C)** Extent of ORO staining quantified by spectrophotometry. Experiments were performed in triplicates in three independent experiments. *p < 0.05, compared with the control groups.

We performed ORO staining to determine the intracellular lipid deposit for OA-induced lipid accumulation (Figure 3). Treatment with OA induced obvious fat deposition in HepG2 cells. The scattered lipid droplet increased the density and size with OA concentration. Considering the excessive deposition of lipids, lipid droplet fused into larger lipid vesicles, or the nuclei were pushed to one side (Figure 3A). The positive staining area of ORO staining showed an increase from 0.12% ± 0.02% to 31.17% ± 1.75% (Figure 3B). The extent of ORO staining quantified by spectrophotometry also confirmed that OA could induce different proportions of intracellular lipid deposition (Figure 3C). The amount of ORO extracted in the 2 and 2.5 mM groups was reduced compared with the 1.5 mM group, and this phenomenon was related to the decrease in the total cell number. The absorbance was significantly higher than the control group (*p* < 0.05). Therefore, the *in vitro* model of lipid accumulation established by OA can simulate the main characteristics of human fatty liver in a dose-dependent manner for further analysis.

### Effects of OA on Actin Cytoskeleton Arrangement

**FIGURE 4:**
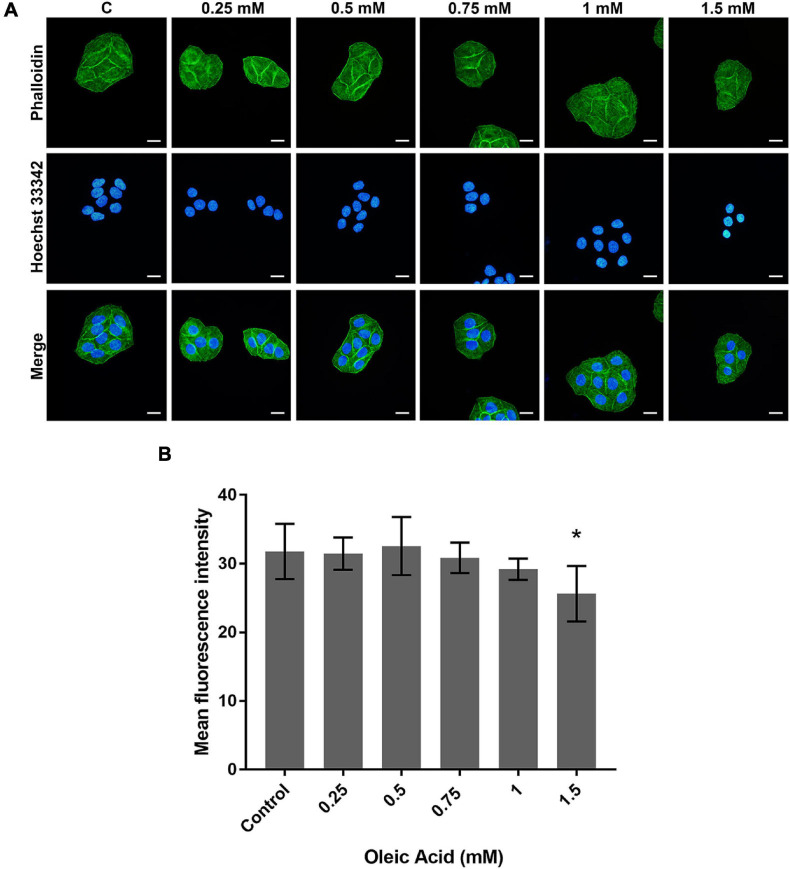
Effects of OA on actin cytoskeleton arrangement for 24 h. **(A)** FITC-labeled Phalloidin was used to stain F-actin. Hoechst 33342 was used to stain nuclei. Images taken at 40 × magnification. Scale bars: 20 μm. **(B)** The mean fluorescence intensity of F-actin. Experiments were performed in triplicates in three independent experiments. *p < 0.05, compared with the control groups.

The actin cytoskeleton arrangement was observed by phalloidin staining (Figure 4). Each group showed parallel bundles of stress fiber in HepG2 cells (Figure 4A). With the increase in concentration, the directional arrangement of the stress fiber remained. The density of actin bundles decreased in the 1.5 mM group, and the nuclei appear as chromatin condensation, suggesting the occurrence of apoptosis. The analysis of fluorescence also showed a weaker intensity in the 1.5 mM group (*p* < 0.05; Figure 4B). Therefore, the actin filaments may not show a marked change within a certain concentration of lipid deposition.

### Effect of OA on the Viscoelastic Response of HepG2 Cells

**FIGURE 5:**
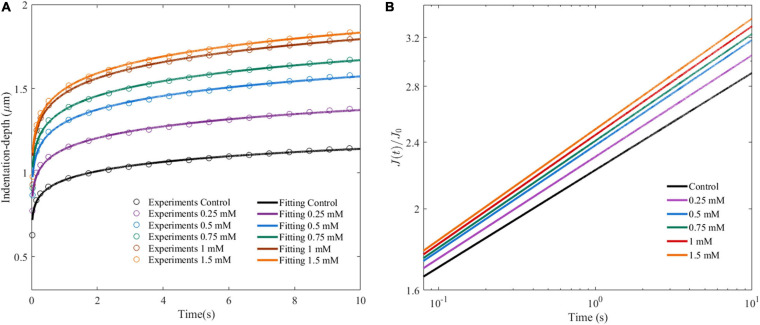
Cell viscoelastic feedback under different concentrations of OA in AFM creep measurements for 24 h. **(A)** Changes of indentation depth as functions of time after treatment of HepG2 cells with different concentrations of OA. The circle marks represent the average of at least 60 indentation tests. Three independent experiments were performed. **(B)** Creep compliance response of HepG2 cells after dimensionless.

Figure 5A shows the average indentation depth of HepG2 cells as a function of time after the given different concentrations of OA. Under the same experimental conditions, the creep compliance increased with OA concentration. The power law model was used to fit the experiment as previously described. Two parameters were obtained, namely, the effective pre-factor compliance (1/*E*_0_) at time *τ*_0_, and the power law exponent *β*. The 1/*E*_0_ and *β*-values showed an increased tendency with lipid accumulation (Figure 5B and Table 1).

**TABLE 1:** Least-square fitting parameters for the power-law model.

Concentration (mM)	1/*E*_0_ (Pa^–1^)	*τ_0_* (s)	*β*
0	1.49E-03	7.53E-05	0.116
0.25	1.84E-03	7.04E-05	0.121
0.5	2.14E-03	6.63E-05	0.126
0.75	2.26E-03	6.33E-05	0.127
1	2.44E-03	6.29E-05	0.130
1.5	2.45E-03	5.96E-05	0.132

## Discussion

Steatosis is recognized in multiple liver diseases and characterized by the deposition of fat droplets (Petäjä and Yki-Järvinen, 2016). The diagnosis for steatosis is important for the early prevention of various liver diseases (Diehl, 2010). In the present work, we used a hepatocyte deposition model with a gradient change to simulate intracellular lipid deposition. In comparison with other fatty acids such as palmitic acid, OA has higher lipid deposition effect and is less damaging (Ricchi et al., 2009). Therefore, OA was selected to amplify lipid deposition and reduce the apoptosis at a certain concentration. The semi-quantitative analysis of ORO staining showed an increased positive area from 3.64 to 19%. The AFM results showed that cell viscoelasticity is obviously concentration-related with lipid accumulation in cells, even when the lipid content is very low. Therefore, viscoelastic parameters can be used to classify light lipid deposition, providing a new insight into the classification of hepatic steatosis, especially in the early stage of the disease.

A significant amount of work has been done in non-invasive diagnostic methods in fatty liver. Yin et al. (2007) demonstrated that softer adipose tissue did not affect the overall stiffness of the liver. The stiffness range of fatty tissue is similar to the normal liver, indicating that the existence of hepatic steatosis in liver tissue does not directly affect the shear stiffness. Barry et al. (2014) found that fat can increase the viscosity of mouse livers, indicating that the viscosity may result in steatosis scoring. Zhu et al. (2015) used gelatin-based phantoms containing different ratios of castor oil to mimic different degrees of steatosis. Their results showed that Young’s modulus decreases, whereas viscosity increases with increasing oil ratio (Zhu et al., 2015). The application of fluorescent probe to visualize intracellular viscosity also proved that fatty liver presents significant fluorescence compared with the healthy liver, indicating the high viscosity in fatty tissue (Yin et al., 2019). Although significant progresses have been made on qualitatively characterizing the mechanical properties changes in liver tissue during hepatic steatosis, both analysis methods and quantitative results are very limited for the accurate evaluation of the status of lipid deposition in single cell level. The present results on the changes of softness and fluidity of HepG2 cells clearly show the quantitative dependence on lipid deposition. This quantitative rule effectively eliminates the interference of the complexity and uncertainty of the mechanical microenvironment of liver tissue, accurately explains the quantitative relationship between the lipid deposition and the changes of observable mechanical indices during fatty liver disease progression, and provides valuable basis for the clinical diagnosis of this disease in the future.

In addition, as the primary structure to maintain the morphology of cells, studies have focused on the mechanical behavior caused by the remodeling of cytoskeleton (Satcher and Dewey, 1996; Rotsch and Radmacher, 2000; Pegoraro et al., 2017) and confirmed the major contribution of actin filament in cell elasticity (Kim et al., 2013; Grady et al., 2016; Sun et al., 2016). However, apart from cytoskeleton and binding proteins, subcellular organelles and crowding protein in cytoplasm may contribute to the rheology of the cells. These components are directly correlated with physiological and pathological process and hard to be solely defined in terms of elastic parameters. Our results showed that the effect of lipid accumulation on the actin might be limited in a certain degree, while mechanical response changes over time. According to soft glassy rheology theory, the degree of internal disorder in the cell can regulate its rheological behavior. Considering that the power-law constitutive relation is adopted, such a rheological behavior can be reflected by the power-law exponent, *β*, as shown in Equation (2) (Kollmannsberger and Fabry, 2011; Efremov et al., 2020). The cells behave more fluid-like when *β* is close to 1 and more solid-like when *β* approaches 0 (Kollmannsberger and Fabry, 2011). To investigate whether the rheological behavior changes with the intracellular environment, we altered the cellular components by adding lipids. Our results showed that the liver cells tend to be more fluid-like as the lipid concentration increases. The deposition of lipids occupied a limited intracellular space, thus possibly leading to aggravated intracellular crowding. This assumption matches the experimental trends in which lipid accumulation increased with *β*.

Our phalloidin staining results revealed that F-actin remained intact within a certain degree of lipid deposition, whereas the values of 1/*E*_0_ and *β* increased with increased lipid deposition. Therefore, the contribution of actin cytoskeleton to the increased 1/*E*_0_ might be limited in our study. The 1/*E*_0_ value increased when the cytoskeleton remained unchanged possibly because of the proportion competition between the “hard-phase” (cytoskeleton) and the “soft-phase” (lipid) in the cells. As the lipid deposition increases, the “soft-phase” becomes increasingly dominant, resulting in the decrease in stiffness and increase in 1/*E*_0_. This finding explains the observed 1/*E*_0_ increase with the OA concentration in a dose-dependent manner. Interestingly, these changes in mechanical properties were mostly caused by the intracellular lipid deposition, rather than the cytoskeleton remodeling, thus providing a new insight into the understanding of the viscoelastic properties of liver cells.

In summary, we discussed the possibility of using viscoelastic feature of single cells to describe the hepatic steatosis. The results showed that the degree of lipid deposition in liver cells was quantitatively correlated with elastic compliance and power-law parameters of the viscoelasticity. The viscoelastic property change in single cells may provide an important potential choice for early hepatic steatosis grading, and the viscoelasticity index might be an accurate detection indicator.

## Data Availability Statement

The raw data supporting the conclusions of this article will be made available by the authors, without undue reservation.

## Author Contributions

JW, RL, and YB conceived the design and wrote the manuscript. JW supervised the study and reviewed the manuscript. RL performed most experiments. RL, YB, and CY analyzed the data. JW and YB contributed to the theoretical analysis. All authors contributed to the article and approved the submitted version.

## Conflict of Interest

The authors declare that the research was conducted in the absence of any commercial or financial relationships that could be construed as a potential conflict of interest.

## Publisher’s Note

All claims expressed in this article are solely those of the authors and do not necessarily represent those of their affiliated organizations, or those of the publisher, the editors and the reviewers. Any product that may be evaluated in this article, or claim that may be made by its manufacturer, is not guaranteed or endorsed by the publisher.
